# The Polygenic Nature and Complex Genetic Architecture of Specific Learning Disorder

**DOI:** 10.3390/brainsci11050631

**Published:** 2021-05-14

**Authors:** Marianthi Georgitsi, Iasonas Dermitzakis, Evgenia Soumelidou, Eleni Bonti

**Affiliations:** 1 1st Laboratory of Medical Biology-Genetics, School of Medicine, Aristotle University of Thessaloniki, 54124 Thessaloniki, Greece; iasonasd@auth.gr (I.D.); esoumelid@auth.gr (E.S.); 2 1st Psychiatric Clinic, School of Medicine, Aristotle University of Thessaloniki, “Papageorgiou” General Hospital, 54603 Thessaloniki, Greece; bonti@auth.gr; 3 Department of Education, School of Education, University of Nicosia, 2417 Nicosia, Cyprus

**Keywords:** specific learning disorder (SLD), dyslexia, dyscalculia, genetic variants, susceptibility

## Abstract

Specific Learning Disorder (SLD) is a multifactorial, neurodevelopmental disorder which may involve persistent difficulties in reading (dyslexia), written expression and/or mathematics. Dyslexia is characterized by difficulties with speed and accuracy of word reading, deficient decoding abilities, and poor spelling. Several studies from different, but complementary, scientific disciplines have investigated possible causal/risk factors for SLD. Biological, neurological, hereditary, cognitive, linguistic-phonological, developmental and environmental factors have been incriminated. Despite worldwide agreement that SLD is highly heritable, its exact biological basis remains elusive. We herein present: (a) an update of studies that have shaped our current knowledge on the disorder’s genetic architecture; (b) a discussion on whether this genetic architecture is ‘unique’ to SLD or, alternatively, whether there is an underlying common genetic background with other neurodevelopmental disorders; and, (c) a brief discussion on whether we are at a position of generating meaningful correlations between genetic findings and anatomical data from neuroimaging studies or specific molecular/cellular pathways. We conclude with open research questions that could drive future research directions.

## 1. Introduction

Specific Learning Disorder (SLD) is a complex disorder with varying manifestations and considerable differences in interpersonal characteristics, albeit present worldwide. According to DSM-5 and the National Joint Committee on Learning Disabilities (NJCLD), SLD is a general term that refers to a group of disorders [1,2,3], which may involve difficulties in reading (dyslexia), written expression (dysgraphia) and/or mathematics (dyscalculia), albeit not accounted for by low intelligence (IQ), sensory acuity (visual problems), poor learning opportunities, or developmental delay (e.g., intellectual disability). Learning disabilities may co-occur with the aforementioned impairments, but are not the result of these conditions [1,4].

The prevalence of SLD varies between 3–12% among the general population, depending on factors such as stringency of measurement cut-offs used for identification [5,6,7], country and level of phonological transparency of the spoken language, sex (male:female ratio 2–3.7:1) [8,9,10], age of assessment, different theoretical perspectives as regards causality, and assessment tools criteria used [6,11]. DSM-5 describes SLD as a neurodevelopmental disorder with a biological origin, which includes an interaction of genetic, epigenetic, and environmental factors. SLD is readily apparent in the early school years in most individuals; symptoms are usually detected when students show a learning profile which is qualitatively lower than their chronological and mental age. However, in some cases, difficulties may become obvious at a later age, when the academic demands rise and exceed the individual’s limited capacities, for example during adolescence or adulthood [2,12]. SLD is a lifelong disorder; its impact can have undesirable outcomes for children, as well as for older individuals, on educational, social, financial and occupational level.

Several studies originating from different scientific fields have tried to investigate the possible causal and/or risk factors of SLD. Neurological-neuroanatomical, biological (genetic, epigenetic), cognitive-information processing, linguistic-phonological, developmental and environmental factors have been incriminated. However, until presently, scientific communities worldwide have not come to an agreement as regards to the exact causes and nature of SLD, neither have they agreed to a commonly accepted definition [13,14,15]. Issues of comorbidity make differential diagnosis an even more complicated task [16]. Arithmetic, reading, or spelling deficits are common in cases with already existing problems in one academic domain compared to the general population [17]; increased dyscalculia rates are observed in families of children with dyslexia [18]. Additionally, dysgraphia rarely occurs alone and frequently co-occurs with dyslexia [19]. Moreover, it is not uncommon for individuals with SLD to show symptoms of Attention-Deficit/Hyperactivity Disorder (ADHD), Specific Language Impairment (SLI), motor-coordination deficiencies, emotional-behavioral difficulties, anxiety, depression, personality disorders, or other conditions; it is not clear whether these conditions comorbid with SLD as simultaneous disorders or are secondary problems deriving from the ongoing academic failure. Nevertheless, each year, a considerable number of children and adolescents as well as adults are referred to diagnostic centers seeking help with their learning difficulties [12,20,21].

From the genetics perspective, SLD is a complex disorder with a strong genetic component; heritability estimates from family and twin studies vary between 40–70% (*h*^2^ = 0.52 for dyslexia and 0.61 for dyscalculia) [22,23,24]. Moreover, reading-related abilities such as word recognition, phoneme awareness, orthographic choice, and phoneme decoding have shown significant heritability estimates above 50% [25]. These high heritability estimates were calculated based on twin studies; a proportion of this genetic component can be attributed to common variants of the human genome, such as single nucleotide polymorphisms (SNPs). According to the latest genome-wide association study (GWAS) on dyslexia, SNP-based heritability yielded an estimate of 20% or 25%, assuming a dyslexia prevalence of 5% or 10%, respectively [26]. The remaining of the genetic risk or “missing heritability” of dyslexia could be potentially explained by other types of genomic variants, such as copy number variants (CNVs) and rare variants. The identification of the latter type of variants requires different methodological and analytical approaches, such as massive parallel deep sequencing, also known as next-generation sequencing (NGS).

Herein, we have synthesized a comprehensive review summarizing our current knowledge on the genetic basis of SLD by compiling data from a significant number of studies. By reviewing the literature from the past 20 years, more or less in chronological order when taking into account the methodological advancements, we inevitably recapitulate the view that underlies most complex neurodevelopmental disorders; the genetic architecture of *Specific* Learning Disorder is not *specific*. We have tried to address this prevailing concept in several aspects: (a) we present the current knowledge on the genetic architecture of SLD and the predisposing factors that are known to underlie specific SLD domains (dyslexia versus dyscalculia) (Section 2 and Section 3); (b) we discuss whether this genetic architecture is unique to SLD or, alternatively, whether there is an underlying common genetic background between SLD and other neurodevelopmental disorders (such as ADHD) (Section 4); (c) we briefly discuss whether we can relate genetic findings with anatomical data from neuroimaging studies or with specific molecular and cellular pathways (Section 5 and Section 6); (d) we conclude with open research questions that could drive future research directions (Section 7).

## 2. Exploring Genetic Susceptibility to SLD—The Early Times

SLD appears to aggregate in families; the relative risk of SLD in reading or mathematics is substantially higher (4–8 times and 5–10 times higher, respectively) in first-degree relatives of individuals with these learning difficulties [1,27,28]. Family history of reading difficulties and parental literacy skills, as well as mathematical difficulties, predict literacy problems or SLD in offspring, indicating the combined role of genetic and environmental factors [1,29,30]. Back when the first efforts to determine the genetic basis of dyslexia started to appear in the literature (Table 1), the disorder was assumed to follow an autosomal dominant inheritance pattern with high, but incomplete, penetrance [31,32]. In the next two decades, it became clear that SLD, and specifically dyslexia, is a complex disorder with marked genetic heterogeneity, as manifested by the identification of at least nine genetic loci spread throughout the genome (Table 1).

Clues into the genetic underpinnings of reading-related traits originally emerged from classical, hypothesis-free, genome-wide linkage screens, linkage analysis in well-phenotyped pedigrees with multiple affected cases, or the detection of rare chromosomal aberrations (mostly translocations) in dyslexic individuals, likely disrupting a susceptibility locus. Owing to the prior view of dyslexia as an autosomal dominant disorder, Online Mendelian Inheritance in Man curates these earlier reports [33]. Briefly, more than nine loci have been identified as candidates for susceptibility to SLD, with several genes, particularly *DYX1C1*, *ROBO1*, *KIAA0319*, and *DCDC2*, repeatedly linked to the disorder and/or measures of reading processes disturbed in dyslexia. Overall, many excellent reviews have covered the earlier efforts to unravel the genetic component of dyslexia [34,35,36,37]. Thus, instead of presenting a redundant text herein, we have compiled the seminal studies that led to the identification of dyslexia-associated genes and loci in Table 1. Apart from the categorical diagnosis, we have also recorded quantitative traits often used as proxies (or endophenotypes) to address the general dyslexia phenotype. This is a common approach successfully used to draw closer to the underlying genetic deficit in complex phenotypes [38]. However, the correlation between these endophenotypes and genetic susceptibility markers is far from optimal, since either the same locus has been associated with different SLD-related traits in different studies [39], or the same quantitative trait has shown marked genetic heterogeneity (Table 1).

Following up on gene mapping, a significant number of studies explored associations between specific variants in candidate susceptibility genes and SLD domains or related traits; we summarize the data in Table 2. Then, for the rest of the review, we focus on the latest advances in the field, considering the shift in the analytical approaches used, driven by the advent of high-throughput genotyping technologies and NGS. We discuss the most recent studies in the text and provide a compilation in Table 3. 

Less is known about the genetics of mathematical abilities or written expression skills, with few genetic studies conducted thus far (Table 1, Table 2 and Table 3). In nearly half of SLD cases, dyslexia and dyscalculia co-occur [40]. This co-occurrence is more frequent than expected by chance and could be partially attributed to shared genetic influences, according to the “generalist genes” hypothesis [41,42]. However, there are still very limited genetic data to support such shared genetic influences [43,44].

**Table 1:** Earlier studies (1993–2013) presenting evidence for association of genomic loci with SLD and/or related traits.

Phenotype Domain/Trait	Locus (Gene(s)) ^1^	Means of Identification	Reference
	**Classical DYX loci**		
Dyslexia/SWR	15q15-q21 (**DYX1**)	Locus-specific linkage analysis	[45]
Severe dyslexia/PA	15q21 (*DYX1C1*)	Chromosomal translocation	[46]
Dyslexia/PA	6p22-p21 (**DYX2**)	Locus-specific linkage analysis	[45]
Dyslexia	6p22 (*KIAA0319*, *DCDC2*)	Linkage analysis and association	[47]
Dyslexia	6p22 (*KIAA0319*)	Linkage analysis and association	[48]
Reading disability	6p22 (*KIAA0319*)	Linkage disequilibrium mapping	[49]
Severe dyslexia	6p22-p21 (*DCDC2*)	Linkage disequilibrium mapping	[50]
Dyslexia/RAN	6p21 (separate from DYX2)	Genome-wide linkage scan	[51]
Dyslexia	2p16-p15 (**DYX3**)	Genome-wide linkage scan	[52]
Dyslexia	2p (**DYX3**)	Locus-specific linkage analysis	[25]
Dyslexia/word- and non-word reading, RAN	2p (**DYX3**)	Locus-specific linkage analysis	[39]
Dyslexia	2p12 (*MRPL19*, *C2orf3*)	Linkage disequilibrium mapping	[53]
Spelling	6q11.2-q12 (**DYX4**)	Genome-wide linkage scan	[54]
PA, naming speed, verbal short-term memory	3p12-q13 (**DYX5**)	Genome-wide linkage scan	[55]
	3p12 (*ROBO1*)	Chromosomal translocation	[56]
SWR, PA (reading-related processes) Dyslexia	18p11.2 (**DYX6**)	Genome-wide linkage scan (QTL-based)	[57]
	18p11.2-q12.2	Locus-specific linkage analysis and association	[58]
	(*MC5R*, *DYM*, *NEDD4L)*		
Dyslexia	11p15.5 (**DYX7**)	Linkage analysis and association	[59]
Severe dyslexia/speech development	1p22	Chromosomal translocation	[60]
Dyslexia	1p36-p34 (**DYX8**)	Chromosomal translocation	[61]
Dyslexia/RAN	1p (**DYX8**)	Locus-specific linkage analysis	[62]
Dyslexia/spelling	1p36-p34 (**DYX8**)	Genome-wide linkage scan (QTL-based)	[63]
Dyslexia/word- and non-word reading, RAN	1p36 (**DYX8**)	Locus-specific linkage analysis	[39]
Dyslexia	Xq27.3 (**DYX9**)	Genome-wide linkage scan	[9]
Dyslexia		SNP-based linkage analysis	[64]
	**Other loci and genes**		
Dyslexia/PD, SWR	21q22.3	FISH/SNP 500k NspI microarray (microdeletion—single family)	[65]
	(*PCNT*, *DIP2A*, *S100B*, and *PRMT2*)		
Dyslexia	15q21.2 *(CYP19A1)*	FISH/SNP genotyping and functional studies	[66]
	(separate from *DYX1C1*)		
Dyslexia	4q13, 16p12, 17q22;	Genome-wide linkage scan	[67]
	suggestive locus at 7q36		
Mathematical (dis)abilities	A score of a set of 10 SNPs in 10 loci, accounting for 2.9% of the variance in math ability	GWAS—Discovery (1200 cases) and validation (2356 cases) cohorts (UK population)	[68]

^1^ Genomic loci as presented in the original corresponding article. SWR: single-word reading, PD: phonological decoding, RAN: rapid automatized naming, PA: phonological awareness, GWAS: Genome-Wide Association Study.

### 2.1. Linkage Screens in Pedigrees

A significant number of dyslexia candidate genes were identified through linkage studies in pedigrees. Reports on familial aggregation of dyslexia, characterized by an autosomal dominant inheritance pattern, continue to become published. These newer reports use a modern approach which combines traditional chromosomal mapping, using dense SNP-based—rather than microsatellite-based—genome-wide genotyping and linkage analysis, coupled with genome-wide gene expression and NGS technologies.

**Figure 1:**
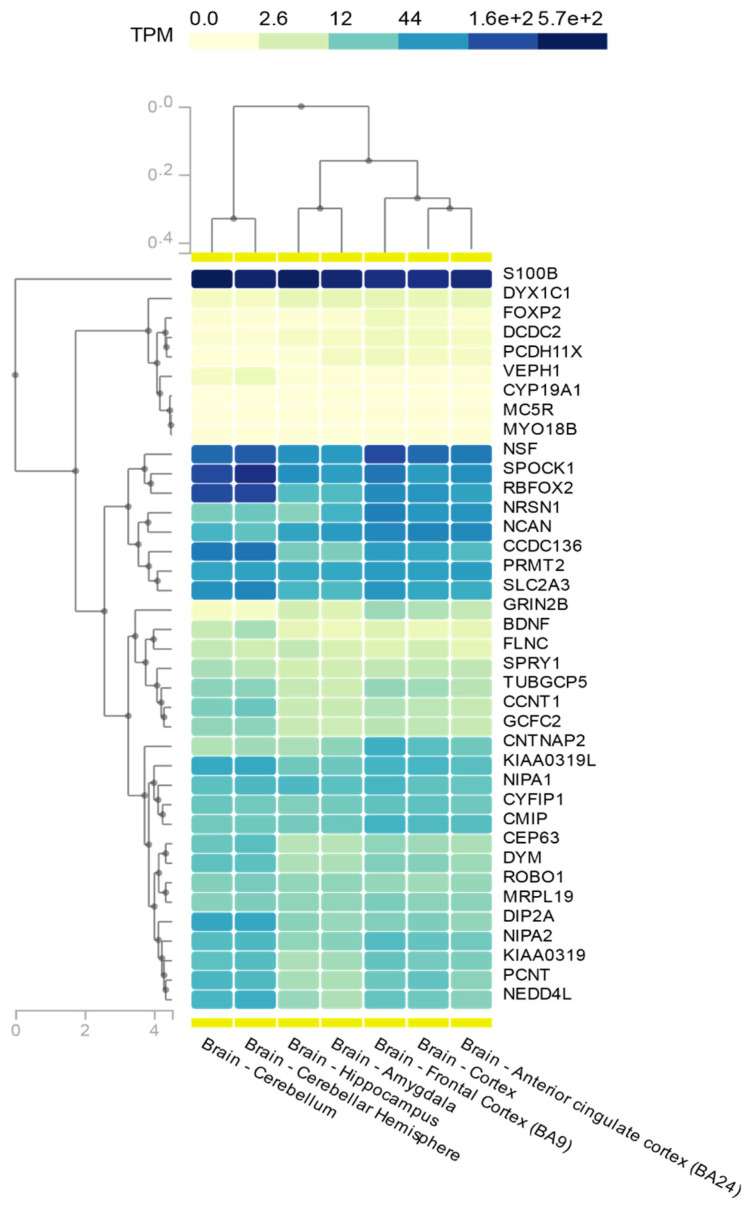
Heatmap of RNA-sequencing-based gene expression from the SLD-associated (protein-coding) genes presented in Table 5, generated in GTEx portal for a multi-gene query in seven brain areas (basal ganglia and hypothalamus are excluded) [204]. *SLC2A3* on chromosome 12 was included as an indirectly associated gene (potentially being *trans*-regulated by a directly associated variant on chromosome 4) (see text in Section 3). *PCDHG* represents a whole gene cluster, thus excluded from the query. TPM: Transcripts per kilobase million (expresses RNA-sequencing reads normalized for gene length and sequencing depth).

For instance, in 2017, Einarsdottir et al. reported the identification of *NCAN* (19p13), a putative novel dyslexia susceptibility gene. It is important, with this example, to highlight that, with the advent of new technologies of greater analytical potential, previously reported families with a clearly defined phenotype, but without a specific genetic diagnosis, can be revisited to offer novel findings. This dyslexia pedigree of Finnish origin, with eight affected cases across three generations [55], was anew subjected to genetic analysis using genotyping and linkage methods, in concert with next-generation whole-exome sequencing (WES) [69]. *NCAN* is expressed in several tissues, including several brain areas (Figure 1); its expression was significantly correlated with that of two other dyslexia candidate genes, namely *GRIN2B and KIAA0319* (Table 2).

An impressive three-generation pedigree of Indian origin was reported by Naskar et al. in 2018; all alive individuals from generation II (*n* = 3), all of their offspring in generation III (*n* = 7) and almost all, but two, of the offspring in generation IV (*n* = 7) were affected with dyslexia in a pattern compatible with autosomal dominant inheritance. Genome-wide SNP genotyping combined with WES revealed several variants in the protocadherin gamma (*PCDHG*) gene cluster (5q31.3) which encodes for alternative *PCDHG* transcripts owing to a large number of alternative 5′ exons. All identified variants clustered in the variable 5′ exons, which encode for the extracellular protocadherin domain. Protocadherins are predominantly expressed in the developing human brain and are known to play a role in neuronal connectivity, thus ensuring formation and maintenance of neural circuits [70].

One of the latest reports of this kind is by Grimm et al., who identified a novel dyslexia-associated gene, namely *SPRY1* (4q28), after studying six out of nine affected individuals across a four-generation pedigree of German origin [71]. SPRY proteins are negative regulators of the Ras/MAPK/ERK pathway but, even though the authors showed that *SPRY1* is expressed in all brain regions, it was not possible to explore the status of mutant *SPRY1* expression in affected cases [71].

### 2.2. Candidate Gene Association Studies

There are two types of candidate gene association studies that have been published during the last two decades regarding genes that underlie genetic susceptibility to reading and mathematical abilities and disabilities. The first approach aims to explore established SLD genes in case-control cohorts of various ethnic origins, typically of Caucasian ancestry. The other approach aims to examine whether genes previously associated with reading and/or mathematical abilities in the general population can be valid in the context of an SLD diagnosis. Table 2 provides an updated list of past and recent publications that followed this study design, while summarizing their major findings.

For the purposes of this review, it is worth highlighting relatively recent studies that employed large sample sizes or were carried out by multicentered cross-linguistic initiatives. For instance, the European consortium NeuroDys performed a cross-linguistic case-control association study of dyslexia with data from more than 950 dyslexic individuals using targeted genotyping of selected markers in *DYX1C1*, *DCDC2*, *KIAA0319*, and the *MRPL19*/*C2orf3* locus [72]. No SNP or haplotype surpassed statistical significance level, and none was associated with dyslexia in samples from more than one population, including populations speaking the same language (e.g., German). This may be potentially explained by differences in diagnosis between countries, genetic architecture heterogeneity among different populations, missing analysis of relevant traits, insufficient power due to the phenotypic heterogeneity of the disease, or combinations of the above [72].

In 2016, Müller and coworkers analyzed 16 SNPs in five genes affecting reading and spelling in the general population, in a German dyslexia case-control cohort [38]. On a single-marker level, no associations survived correction for multiple testing, but the observed risk alleles in *KIAA0319* were in agreement with associations from both the general population, as well as other dyslexia studies [72]. No gene-specific haplotypes were associated with dyslexia in *KIAA0319*, *DYX1C1*, or *DCDC2*. When performing polygenic analysis, an increased number of risk alleles was observed within dyslexic cases compared to controls. The authors also demonstrated by in silico analyses on publicly available eQTL data that the SNPs residing in *DCDC2*, *KIAA0319* and *DYX1C1* affect the corresponding genes’ expression, as well as the expression of a gene in the vicinity of *DCDC2*, namely *MRS2* [38].

In the study of Sánchez-Morán et al., the authors explored associations of three variants, one in each of three established dyslexia genes in 286 dyslexic children versus 1197 controls. Again, no single-marker association reached statistical significance, but pairwise SNP interaction between rs2274305 in *DCDC2* and rs4504469 in *KIAA0319* showed significant association with dyslexia as well as with dyslexia plus comorbid ADHD. In addition to the case-control design, these candidate SNPs were also associated with cognitive traits in the general population (*n* = 3357): rs2274305-*DCDC2* with phoneme awareness (PA) and rapid automatized naming (RAN), *DYX1C1* with word-reading and RAN, and rs4504469-*KIAA0319* with word-reading, RAN, and syllable discrimination [73]. *DCDC2* and *KIAA0319* reside on the same locus, yet they are not in linkage disequilibrium; this points to independent, but synergistic, association, since a *DCDC2* risk haplotype interacts synergistically with a *KIAA0319* haplotype, conferring higher risk in reading disability when both risk haplotypes occur together rather than separately [74].

A large cohort of more than 1500 unimpaired individuals was recently analyzed for genetic variants across 14 genes previously associated with dyslexia. Doust et al. performed gene-set-based analysis for reading impairment candidate genes and for the Gene Ontology biological pathway genes for ‘axon guidance’ and ‘neuron migration’. The lack of replication of previous associations in this carefully characterized, yet unselected for SLD/dyslexia, cohort could be true or could be attributed to a number of other reasons: lack of statistical power to detect variants of small effect size, despite being one of the largest cohorts analyzed for reading abilities thus far, or sampling bias owing to participants’ recruitment from a twin registry [75].

The abovementioned studies are used as examples to illustrate that despite their undoubtedly careful design, statistically significant associations were still not reached or were, at best, nominal. Improvements, such as the incorporation of much larger numbers than past candidate gene studies and the recruitment of extremely carefully scrutinized participants across a number of reading and mathematical traits, left much of the genetic susceptibility puzzle of a common disease-common variant hypothesis unanswered. The field had to move on to hypothesis-free approaches; this advancement is reviewed in the following section.

**Table 2:** Summary of association studies of established or candidate SLD/dyslexia genes.

Phenotype (Trait/Subphenotype)	Gene(s)	Variant(s) Associated with Phenotype or Trait	Sample Size and Study Design	Reference
**Genes Residing in Classical DYX Loci**				
Dyslexia/PA, RAN, and other traits	*DYX1C1*	rs11629841 and haplotypes of rs11629841 with rs3743204 and rs692691	148 nuclear families (470 individuals)	[76]
Dyslexia	*DYX1C1*	No association	264 nuclear families (1153 individuals)	[77]
Dyslexia	*DYX1C1*	c.1249G>T coding variant	191 trios	[78]
Dyslexia/short-term memory	*DYX1C1*	c.−3G>A and c.1249G>T minor alleles haplotype	212 nuclear families (677 individuals)	[79]
Dyslexia/short-term memory	*DYX1C1*	rs3743205/rs3743204/ rs600753 haplotype in females	366 trios	[80]
Reading ability (reading and spelling traits)	*DYX1C1*	rs17819126 coding variant	284 DZ twins, 164 DZ twin families, 143 MZ twin families	[81]
Dyslexia/Reading ability (12 cognitive traits)	*DCDC2*	10/31 SNPs in *DCDC2*	153 nuclear families (536 individuals)	[82]
Dyslexia	*DCDC2*	No association	396 trios	[83]
Dyslexia (severe versus non-severe)	*DCDC2*	rs793862, rs807701, rs80772 and intron-2 deletion	72 cases/184 controls	[84]
Reading ability (7 reading and spelling traits)	*DCDC2*	21 SNPs of which rs1419228 was associated with poorer general reading performance	522 twin families (1067 individuals) (unselected population)	[85]
Dyslexia/word-reading and spelling	*DCDC2*	rs793862 and rs807724 minor alleles in SLD or comorbid cases	225 cases/442 controls (plus 54 comorbid SLD/SLI/ADHD cases)	[86]
Dyslexia and mathematics (numerical facts and mental calculation)	*DCDC2* and *DYX1C1*	c.−3G>A, c.1249G>T in *DYX1C1* and intron-2 deletion/STR in *DCDC2*	180 nuclear families (581 individuals)	[87]
Dyslexia/6 traits of reading ability	*DCDC2*	Intron-2 STR alleles associated with word- and non-word repetition	303 nuclear families (973 individuals)	[88]
Dyslexia	*DCDC2*	14 SNPs of which several SNPs and two haplotypes were associated under different models	196 cases/196 controls	[89]
Dyslexia/6 traits of reading ability	*DCDC2* and *KIAA0319*	5 SNPs within *KIAA0319* Pairwise associations between a *DCDC2* and a *KIAA0319* variant	264 nuclear families 350 cases/273 controls	[90]
Reading abilities (5 reading and spelling traits)	*KIAA0319*	rs2143340 associated with poor reading and spelling	~6000 individuals	[91]
Dyslexia/6 traits of reading ability	*KIAA0319*	rs9461045 associated with dyslexia traits	264 nuclear families (of which 126 comprised a severity sample)	[92]
Dyslexia/Reading, spelling, and phonological traits	*DCDC2* and *KIAA0319* *NRSN1*	rs6935076 in *KIAA0319* associated with dyslexia and spelling and 3 SNPs in *NRSN1*	291 nuclear families (of which 165 are trios)	[93]
General reading abilities (word-reading and spelling)	*KIAA0319* and *CMIP*	rs2143340 in *KIAA0319* and rs6564903 in *CMIP*	225 cases/442 controls (plus 54 comorbid SLD/SLI/ADHD cases)	[86]
Dyslexia and mathematics	*ROBO1*	rs333491 associated with mental calculation accuracy	179 nuclear families (of which 154 comprised a severity sample)	[94]
Dyslexia Word-reading efficiency and RAN	*KIAA0319L* *KIAA0319L*	rs7523017 associated with dyslexia A four SNP-haplotype	291 nuclear families 156 nuclear families	[95]
**Other dyslexia-candidate genes**				
Dyslexia/6 traits of reading ability	*CNTNAP2*	rs2710102 associated with non-word repetition	188 trios	[96]
Dyslexia/6 traits of reading ability	*FOXP2*	rs7782412 major allele associated with non-word repetition and real-word reading efficiency	188 trios	[96]
Dyslexia (mismatch response)	*SLC2A3*	rs4234898 on chromosome 4 associated with mismatch response	200 cases (discovery set) 186 cases (replication set)	[97]
Dyslexia/IQ and cognitive processes and mathematics	*GRIN2B*	rs5796555 and rs1012586 associated with dyslexia	466 nuclear families, of which 227 comprised a severity sample	[98]
Reading ability (reading comprehension, phonological memory)	*BDNF*	rs6265 associated with poorer reading performance rs6265 associated with increased brain activity in areas contributing to phonological and reading competence	81 children 94 children	[99] [100]
**Dyslexia-associated gene panels**				
Dyslexia/word-reading and spelling	*DYX1C1*, *DCDC2*, *KIAA0319*, and *MRPL19*/*C2orf3* locus	No association	958 cases/1150 controls	[72]
Dyslexia	*MRPL19*, *C20RF3*, *ROBO1*, *DCDC2*, *KIAA0319*, *DYX1C1*, *CNTNAP2*, *ATP2C2* and *CMIP*	rs807724 in *DCDC2* associated with dyslexia	331 cases/maximum 363 controls	[101]
Dyslexia/spelling	*CYP19A1*, *DCDC2*, *DIP2A*, *DYX1C1*, *GCFC2 (C2orf3)*, *KIAA0319*, *MRPL19*, *PCNT*, *PRMT2*, *ROBO1* and *S100B*	A non-synonymous SNP in *DCDC2* (rs2274305) and a non-coding SNP in *S100B* (rs9722) associated with dyslexia	361 cases/261 controls 575 affected, 376 unaffected and 511 of unknown status (family-based)	[102]
Dyslexia	*DYX1C1*, *DCDC2*, *KIAA0319*, *ROBO1* and *TDP2*	Nominal associations only (rs7765678 in *DCDC2*, rs2038137 and rs6935076 in *KIAA0319*)	383 cases/357 controls	[38]
Reading abilities (Word/Non-word reading fluency, PA, RAN)	*Top hits from previous GWAS on reading (SLD) and language (SLI) (dis)abilities*	No association	307 nuclear families (483 children/505 adults)	[103]
Reading ability	*CYP19A1*, *DCDC2*, *DYX1C1*, *GCFC2* (*C2orf3*), *KIAA0319*, *MRPL19*, *ROBO1*, *KIAA0319L DIP2A*, *PRMT2*, *PCNT*, *S100B*, *CNTNAP2* and *CMIP*	No single-marker association 62 SNPs—Gene-based SNP-set associations were significant for *DYX1C1*, *DIP2A*, *CYP19A1*	1217 old adults (>70 yrs) (unimpaired)	[104]
Dyslexia Word reading, RAN, and syllable discrimination	*KIAA0319*, *DCDC2*, and *DYX1C1*	No single-marker association Pairwise SNP association with dyslexia (rs2274305 in *DCDC2* and rs4504469 in *KIAA0319*) rs2274305 in *DCDC2* rs57809907 in *DYX1C1* rs4504469 in *KIAA0319*	286 cases/1197 controls 3357 individuals (total cohort)	[73]
Reading and spelling ability	*CMIP*, *CNTNAP2*, *CYP19A1*, *DCDC2*, *DIP2A*, *DYX1C1*, *C2orf3*, *KIAA0319*, *KIAA0319L*, *MRPL19*, *ROBO1*, *PCNT*, *PRMT2* and *S100B*	No association (>9500 SNPs and gene-based SNP-sets)	1505 individuals (unimpaired)	[75]
**Other SLD domains**				
Reading and mathematical traits indicative of dyslexia and dyscalculia, respectively	15q11.2(BP1-BP2)—*TUBGCP5*, *NIPA1*, *NIPA2*, *CYFIP1*	15q11.2(BP1-BP2) deletion CNV associated with worse outcome in reading and mathematical abilities	167 controls, carriers of neuropsychiatric CNVs	[43]
Dysgraphia	*DCDC2*, *DYX1C1*, *KIAA0319* and *ROBO1*	rs3743204 in *DYX1C1* and rs793842 in *DCDC2* associated with dysgraphia measurements	21 cases/18 controls	[105]

PA: phonological awareness, RAN: rapid automatized naming, SNP: single nucleotide polymorphism, cases = dyslexic cases, controls = unimpaired individuals, DZ: dizygotic (twins), MZ: monozygotic (twins), STR: short tandem repeat.

## 3. High-Throughput Genome-Wide Analysis Continues to Shed Light on the Genetic Architecture of SLD

### 3.1. Genome-Wide Association Studies (GWAS) and Polygenic Risk Scores (PRSs)

GWA studies are not hypothesis-driven, unlike candidate gene association studies that are designed with specific questions in mind, interrogating particular genes or genomic loci implicated in specific molecular pathways or biological processes hypothesized to be involved. Nevertheless, GWAS proved less successful than originally expected in helping to pinpoint SLD susceptibility loci, partly owing to the heterogeneous dyslexia phenotype and diagnostic/recruitment criteria used or to the small sample numbers analyzed compared to other neurodevelopmental/psychiatric phenotypes. Small sample sizes confer low detection power for common variants with small effect sizes, especially considering the stringent statistical correction for multiple testing over hundreds of thousands or millions of variants that needs to be taken into account. To compensate, genome-wide screening of the general population for DNA variants associated with reading, arithmetic and language abilities as heritable traits attracted intense research interest; these were viewed as ”intermediate phenotypes”, or quantitative traits acting as endophenotypes, determined by a genetic background that potentially also underlies SLD etiology.

Reading skill as a quantitative trait was explored for the first time by applying a GWAS approach using the extremes of its continuous distribution. Two groups, low versus high reading ability, comprising a total sample of 1500 children, were genotyped using a low-density SNP microarray (~100 k). Top candidate SNPs showing the largest allele frequency differences between extreme-ends groups were validated in an independent sample of 900 age-matched children. Of those, ten SNPs showed nominally significant association with continuous variation in reading ability [106]. Since this seminal effort, a significant number of studies have been conducted, several of which focused on variants with pleiotropic effects in both reading and language traits (Table 3) [107,108,109]. We believe that the most recent one deserves highlighting for two reasons. First, the authors studied reading disability predictors, namely RAN and rapid alternating stimulus, in a sample of more than 1300 Hispanic-American and African-American young individuals. Second, they found, for the first time in a GWAS design, genome-wide significance for a variant located on the upstream region of a long non-coding RNA (lncRNA) gene, namely RPL7P34, 30kb upstream of RNLS (10q23.31). It was suggested that this variant resides on an enhancer element that potentially interacts with an active RNLS transcription start site in the hippocampus, owing to chromatin’s three-dimensional structure. The variant was further associated with structural variation (cortical volume) in the right inferior parietal lobule of an independent multi-ethnic sample [110]. Currently, it remains largely unknown how non-coding regions of the genome may impact reading traits; the identification of variants in gene regulatory regions, as recently demonstrated for *ARHGEF39* in SLI [111], or the role of post-transcriptional (e.g., miRNA-based) regulation of gene expression, is undoubtedly an exciting new field of research.

Coming to the context of dyslexia, one of the first GWAS, albeit of a very small scale in comparison to current standards (200 cases for discovery and 186 for replication, tested for a limited number of markers (300k)), identified rs4234898 on chromosome 4 as a trans-acting regulatory variant for *SLC2A3* which resides on chromosome 12. *SLC2A3* codes for a glucose transporter in neurons, and its reduced expression in lymphoblastoid cell lines was shown to be significantly associated with the minor rs4234898 allele. It was suggested that *SLC2A3* might act as a susceptibility gene for an electrophysiological endophenotype in dyslexic children with glucose transport deficits, namely mismatch negativity (MMN) or mismatch response. MMN serves as a measure for speech perception and automatic speech deviance which has been found impaired in dyslexic children [97]. This mismatch response endophenotype was later shown to associate with common variants in *DYX1C1* [112], unlike common variants in *DCDC2* and *KIAA0319* [113].

The largest GWAS for dyslexia-specific traits was recently published, with data generated for almost 3500 reading-impaired and typically developing children of European ancestry from nine countries speaking six different languages. Genome-wide significance was observed with RAN for four variants on 18q12.2, within *MIR924HG* (rs17663182), and a suggestive association on 8q12.3 within *NKAIN3.* It is of note that *MIR924* is predicted to regulate candidate dyslexia susceptibility genes like *MRPL19* and *KIAA0319L*, as observed via in silico analysis of putative miR-924 binding sites [114]. The same group performed a polygenic risk score (PRS) analysis between eight reading traits and different neuropsychiatric disorders (ADHD, ASD, major depressive disorder and schizophrenia), educational attainment, and neuroimaging phenotypes (seven brain areas) and found a significant genetic overlap between some of these reading traits and educational attainment and, to a lesser extent, with ADHD [114]. This initiative led to an even larger dyslexia case-control GWAS of almost 2300 cases and 6300 controls, a subset of which overlapped with the same authors’ 2019 paper [26]. No novel genome-wide significant associations emerged at single-marker level; gene-based analysis from the top SNP association signals revealed *VEPH1* (3q25) as a top candidate gene, but no specific pathways showed significant enrichment [26].

Actually, the first study assessing the reading ability of non-dyslexic children and adolescents with the use of PRS analysis was published in 2017. The authors in this study utilized GWAS data from >5800 cases and used educational attainment (=years of education completed) to predict reading performance in English. They calculated a PRS-heritability estimate of reading ability of almost 5%, based only on common variants. This estimate represents approximately 7% of the total heritability for reading ability (h^2^ = 70%; 5%/70%) evaluated through twin studies [115]. However, if calculating the PRS-heritability estimate using an SNP-heritability estimate, which was shown to account for 22% of the total genetic variance [116], then the PRS-heritability estimate can explain a significant 23% (5%/22%) of the genetic variance observed for reading ability, an estimate that remained significant after accounting for age-specific cognitive ability and family socioeconomic status [115].

The use of PRSs is a rather young addition to the armor of (statistical) tools to evaluate the genetic component of complex traits, even more so for complex cognitive skills like reading performance; yet, we can already foresee its potential. Given its inherent nature (as DNA variants do not change by age), knowing the individual genetic differences in reading ability perhaps may prove useful in the early prediction of reading problems like dyslexia. This will require large multicentered initiatives of tens of thousands of participants. However, because language transparency is an important issue in assessing dyslexia, perhaps large GWAS with participants using the same language would be powerful enough to explore the applicability of PRS further, an approach already tested by Gialluisi et al. in their 2019 analysis [114].

The first GWAS study conducted to exclusively assess mathematical ability and disability was published ten years ago; two groups of children from the Twins Early Development Study, with high versus low mathematical ability (600 individuals per group), served as the discovery cohort, and 2356 individuals, spanning the entire distribution of mathematical ability, were used for validation purposes. Out of 10 top candidate SNPs, rs11225308 (*MMP7*), rs363449 (*GRIK1*), and rs17278234 (*DNAH5*) were the variants most significantly associated with mathematical ability. Because the effect sizes of these 10 SNPs were small, the authors created an ‘SNP-set score’ for each of the 2356 individuals, which accounted for 2.9% of the variance in their sample [68]. In fact, by using this SNP-set score, it was shown that one third of children who harbored ≥50% of the identified risk alleles were nearly twice as likely to be in the lowest-performing 15% of the mathematical ability distribution [68]. This score was later correlated with certain environmental factors, demonstrating likely gene × environment interactions [117].

Subsequently, in a sample of almost 700 dyslexic cases and more than 1400 controls, available GWAS data were reanalyzed to associate genetic variation specifically with dyscalculia. The authors found rs133885 in *MYO18B* to be strongly correlated with mathematical abilities in the dyslexia sample and, to a lesser extent, the general population. A significantly lower depth of the right intraparietal sulcus, an anatomical brain region involved in numerical processing in humans, was associated with rs133885 [118]. However, this association was not supported in the subsequent analysis of a much larger collection of 5144 individuals from four cohorts of European ancestry, 329 of which were diagnosed with dyslexia [119]. A third GWAS aiming to explore the genetic contributions to mathematical ability was conducted in a general population sample of 602 adolescents/young adults with excellent verbal ability but either high or low mathematical ability. The marker with the largest effect size was rs789859, located in the promoter of *FAM43A* and in high linkage disequilibrium with two SNPs in the adjacent *LSG1* gene (3q29), a region previously linked to learning difficulties and autism [120]. Although the encoded protein’s function remains obscure, *FAM43A* was found expressed in the brain, cerebellum and spinal cord [120].

One GWAS was conducted exclusively on the purpose to assess mathematical ability in the general population of Chinese elementary school students in 2017. Two discovery and one replication groups were used, totaling almost 1600 individuals. Sample meta-analysis revealed four linked SNPs in *SPOCK1* associated on a genome-wide significance level with a decrease in math scores on two examination periods [121]. Interestingly, mutations in *SPOCK1*, which encodes for the extracellular proteoglycan testican-1, have been associated with ID and microcephaly in humans, whereas *Spock1* mouse models have demonstrated strong gene expression in the brain as well as its role in neurogenesis [121].

By now, it has become clear that because GWAS are designed to target common variants, often in non-coding, regulatory or even intergenic regions, they do not necessarily directly reveal the true effect of likely pathogenic variants, as it would be expected in the case of rare coding variants. On the other hand, initial genome-wide genotyping platforms were designed based on Caucasian genome frequencies and most of what we currently know about reading and mathematical abilities and disabilities originates from studies of individuals of Caucasian ancestry, despite the fact that SLD affects populations globally and irrespective of language. Thus, we are largely unaware of the genetic architecture of SLD *across* populations and ethnic ancestries. GWAS, despite setting the grounds for unbiased genome-wide interrogations, most often than not, have returned results that could be hardly replicated. This has been attributed either to small effect sizes of common variants, especially for quantitative traits such as reading-associated traits, small sample sizes to reveal statistically powerful associations or even to lack of consensus in SLD diagnosis. Hence, alternative yet complementary methods, as those described in the next paragraphs, have significantly contributed in the delineation of the genetic architecture of SLD during the last years.

### 3.2. Copy-Number Variants (CNVs)

Part of the missing heritability of SLD may be also caused by structural variants. CNVs have been extensively explored in other neurodevelopmental disorders, such as ASD, ID [122,123,124], Tourette Syndrome [125,126], and SLI [127]; results for SLD have been inconclusive. On one hand, recent analyses of dyslexia cohorts indicate that rare, large CNVs may not confer a significant burden [122,128]. On the other hand, rare de novo or inherited deletions or duplications, such as the Xq21.3 region bearing *PCDH11X* [129], 17q21.31 harboring *NSF* [130], and 15q11.2(BP1-BP2) harboring four highly conserved genes (Table 3) [43,44], have been reported in cases with SLD. Earlier, a father and his three affected sons were found to carry a submicroscopic deletion (at least ~176 kb) on 21q22.3, encompassing the 3′ region of *PCNT*, genes *DIP2A* and *S100B* and the 5′ upstream sequence of *PRMT2*. The deletion perfectly segregated with dyslexia and standard scores for phonological decoding and single-word reading of below −1.5 to −2 standard deviations [65]. As described later (Section 3.3), a non-coding variant in *S100B* was also associated with spelling performance in a German family set [102].

Different loci have been found to harbor deletions and duplications in patients with various clinical presentations and comorbid math comprehension difficulties. Children with the 22q11.2 deletion syndrome show considerable difficulties in procedural calculation and word problem solving due to difficulties in understanding and representing numerical quantities, despite relatively normal reading performance [131]. A 22q11.2 deletion spanning LCR22-4 to LCR22-5 interval was found in an 11-year-old girl with normal intelligence, number sense deficit, normal results in spelling and reading tests and social contact difficulties [132]. A severely affected girl with X-linked myotubular myopathy and math difficulties was found to carry an inherited 661kb Xq28 microduplication with a skewed X chromosome inactivation pattern [133]. If we exclude syndromic cases, reports on individuals presenting exclusively with mathematical impairments who bear rare or novel de novo or inherited CNVs are truly scarce. An increase of CNVs of the Olduvai protein domain on 1q21 (*NBPF15*), previously known as DUF1220, appear to be involved in human brain size and evolution and may determine the mathematical aptitude ability of both sexes [134]. This genetic locus is highly expressed in brain regions with high cognitive function [135], but it has not been studied in the context of mathematical disabilities.

Last but not least, a recent study from the Icelandic population investigated the effect of 15q11.2(BP1-BP2) deletion in cognitive, structural and functional correlations of dyslexia and mathematical disabilities. This CNV was previously associated with cognition deficits in non-neuropsychiatric cases with a history of SLD [43]. Later, Ulfarsson et al. showed that the deletion conferred high risk in either dyslexia or dyscalculia, but the risk was even higher in the combined dyslexia plus dyscalculia phenotype; all deletion carriers performed worse on a battery of tests assessing reading and mathematical abilities. In the same sample, structural magnetic resonance imaging (sMRI) and functional MRI (fMRI) were performed, demonstrating that smaller left fusiform gyrus and altered activation in the left fusiform and left angular gyrus also associated with the 15q11.2 deletion [44]. These brain areas are involved in the retrieval of mathematical facts, the usage of learned facts and the performance of arithmetic operations [136,137,138]. This anatomical and functional brain differentiation could be one cause of the greater risk observed for the combined phenotype in deletion carriers.

Either de novo or transmitted, these structural variations may produce a yet unknown spectrum of disturbances on genomic, transcriptomic and proteomic level, for instance haploinsufficiency in the case of deletion or overexpression in the case of duplication [139,140], consequently also affecting subsequent protein-protein interactions; these are hypotheses that warrant further investigation. Interestingly, the 15q11.2(BP1-BP2) duplication carriers do not show significant cognitive impairments, compared to 15q11.2(BP1-BP2) deletion carriers, and are comparable to no-CNV controls [44]. This fact supports the role of haploinsufficiency for the genes mapped on this region, particularly *CYFIP1*, which was shown to be involved in neuronal development [141].

### 3.3. Next-Generation Sequencing

It is unclear how much of the missing heritability of SLD could be attributed to rare or de novo variants of moderate or high effect, even though this issue has been extensively studied with respect to ID, ASD and developmental delay [142,143,144]. With the emergence of NGS technology, the identification of rare variants could help fill in some of the missing pieces of the puzzle. Sequencing data have only recently begun to emerge for SLD, supporting the influence of certain genomic regions on reading performance and related disabilities. As expected, the first efforts concentrated and sources were allocated on the validation of previously established or suspected dyslexia genes in various populations.

Originally mapped through a submicroscopic deletion on 21q22.3 in a dyslexia family [65], *S100B* was one of 11 genes to be scrutinized for rare variants using targeted NGS in more than 900 dyslexia cases from Finland and Germany; a 3′ UTR variant (rs9722), located on or adjacent to in silico predicted miRNA target sites, was associated with spelling performance in the German family set. Moreover, a nonsynonymous variant in *DCDC2* (rs2274305) was associated with severe spelling deficiency in the same sample set [102]. A similar approach was applied to a subsequent next-generation targeted sequencing effort by Adams et al., who selected dyslexia-associated candidate genes to be screened in 96 affected, unrelated subjects of European ancestry from the Colorado Learning Disability Research Center (CLDRC). These cases were selected based on a CLDRC-derived discriminant score indicating impairment in reading ability [145]. The authors searched for rare, likely disrupting, variants and calculated a statistically significant increase in the frequency of observed mutations in dyslexia cases—compared to data from 1000 Genomes Project—in two loci: 7q32.1 harboring the adjacent genes *CCDC136* and *FLNC* (19 missense variants) and 6p22 harboring *DCDC2* and *KIAA0319* (74 missense variants). The data indicate that these regions must have an influence on reading performance, even though not all of the above-mentioned genes show detectable expression in the brain (Figure 1) [145].

The first whole-exome sequencing (WES) study was published in 2015 by Einarsdottir et al. in an effort to identify the genetic basis of a familial form of dyslexia with likely complete penetrance in an extended three-generation pedigree with 12 confirmed dyslexic and four uncertain cases. Through several filtering steps on WES data, a small heterozygous in/del variant was identified in *CEP63*, namely c.686–687delGCinsTT; its transmission was compatible with autosomal dominant inheritance. This rare variant codes for a non-synonymous change in a highly evolutionarily conserved amino acid (p.R229L), which was in silico predicted to alter the protein’s tertiary structure [146]. As discussed later (Section 6), CEP63 is a centrosomal protein involved in microtubule organization and, even though it is ubiquitously expressed (Figure 1), brain-specific isoforms may be affected by such rare variants. It still remains to be seen whether *CEP63* variants are linked to dyslexia in additional cases. 

Several other reports have also demonstrated that dyslexia-associated genes encode proteins with structural and functional roles in cilia (Section 6) [147,148,149,150,151,152,153]. Recently, rare variants were identified in two genes related to motile cilia structure and function, namely dynein axonemal heavy chain 5 (*DNAH5*) and dynein axonemal heavy chain 11 (*DNAH11*). This represents the first whole-genome sequencing (WGS) analysis in literature of two unrelated dyslexia cases, with situs inversus and ADHD symptomatology [154]. Even though direct links between visceral and functional brain asymmetry are lacking, visceral asymmetry (e.g., situs inversus) is comorbid, at least in some cases, with psychiatric and neurodevelopmental disorders [155]. Although it could not be proven unequivocally that the identified variants in *DNAH5* and *DNAH11* cause susceptibility to dyslexia, these two genes represent good candidates for further studies.

Overall, the most recent studies that have used state-of-the-art methodology to look for either likely pathogenic CNVs or rare variants in isolated families have provided clues for the implication of novel genes. Family-based studies continue to be a powerful method to unravel the genetic basis of dyslexia [146]. However, variations in reported loci do not explain, so far, but a small percentage of the genetic component of SLD. Consequently, much of the heritability of learning-related disorders remains unaccounted for. Perhaps the answer is not “hiding” exclusively in single, rare variants that remain yet to be identified, but also in gene × gene and higher-order chromatin interactions or epigenetic regulatory mechanisms and ways that the environment can determine the (epi)genome [156]. It is of note that epigenome-wide association studies have not been reported yet.

**Table 3:** Recent studies (2013–2021) reporting novel genomic loci and genes associated with SLD and related traits using high-throughput methodologies.

Phenotype (Trait/Subphenotype)	Gene(s)	Experimental Approach	Reference
Reading abilities (reading, spelling)	Suggestive associations only	GWAS (meta-analysis)	[108]
Dyslexia or Dyslexia+SLI comorbidity	*ZNF385D* (comorbid cases only)	GWAS (case-control)	[107]
Dyslexia (phonological coding skill)	Suggestive linkage and suggestive associations only	GWAS (case-control)	[67]
Dyslexia	*PCDH11X*	CNV + SNP microarray (11 families)	[129]
Dyslexia/Dyscalculia	15q11.2(BP1-BP2) harboring *TUBGCP5*, *NIPA1*, *NIPA2* and *CYFIP1*	Targeted CNV and neuroimaging analysis	[43,44]
Reading abilities (reading, spelling, phonological awareness)	*RBFOX2*, *CCDC136*/*FLNC*	GWAS (meta-analysis)	[109]
Dyslexia	*NSF*	CNV + SNP microarray (10 families)	[130]
Dyslexia	*CEP63*	WES (single family)	[146]
Dyslexia	*S100B*	Targeted NGS (11 genes panel)	[102]
Dyslexia	*CCDC136* and *FLNC*	Targeted NGS—11 loci harboring 25 genes	[145]
Dyslexia	*NCAN*	SNP microarray and linkage analysis, WES (single family)	[69]
Dyslexia	*PCDHG* gene cluster	SNP microarray and WES (single family)	[70]
Dyslexia/8 cognitive traits	*MIR924HG* (associated with RAN)	GWAS (case-control)	[114]
Dyslexia	*VEPH1* (gene-based analysis)	GWAS (case-control)	[26]
Dyslexia	*SPRY1*	SNP microarray and linkage analysis (single family)	[71]
Reading ability (word reading)	*LINC00935* and *CCNT1*	GWAS (case-control)	[157]
Mathematical abilities	*MYO18B*	GWAS (case-control)	[118]
Mathematical abilities	rs789859 intergenic to *LSG1* and *FAM43A* (3q29)	GWAS (high versus low mathematical ability)	[120]
Mathematical abilities	*SPOCK1*	GWAS (meta-analysis)	[121]

SLI: specific language impairment, GWAS: Genome-Wide Association Study, WES: whole exome sequencing, CNV: copy number variant, SNP: single nucleotide polymorphism.

## 4. Comorbidity and Genetic Correlation with Other Neurodevelopmental Phenotypes

Since the “generalist genes” hypothesis was proposed [41], it has become common ground, and recent emerging evidence also supports, that neurodevelopmental disorders share, to a certain extent, a common genetic background. High-impact studies support the pleiotropic or even antagonistic actions of genes and their variation on complex phenotypes, with a particular focus on psychiatric disorders [158,159]. Cross-disorder analyses aim at identifying transdiagnostic variants that could point eventually toward common underlying traits (e.g., cognitive, imaging), molecular pathways, and even symptoms or environmental risk factors [160]. Pleiotropy is mainly manifested via loci harboring genes that show brain-specific expression; thus, these genes are expected to be particularly important in neuronal development, with potential implications for better disease classification and management or future treatment interventions. Prominent examples in the field include schizophrenia and bipolar disorder [161], ASD and ADHD [162,163], Tourette Syndrome (TS) and Obsessive–Compulsive Disorder (OCD) [164,165], and, more recently, OCD and anorexia [166], or TS and ADHD/ASD [167].

As highlighted in the introduction, individuals with SLD show symptoms of ADHD, SLI, or other conditions, but it remains unclear whether these comorbid with SLD or are secondary problems deriving from the impairments caused by SLD. Reading and language are both viewed as highly heritable traits that are likely to share common genetic and/or neurobiological influences [168]. Shared genetic contributions between reading and language performance have been explored in several studies using candidate gene association analyses or GWAS meta-analysis [101,103,108,109]. For instance, Luciano et al. found strong associations with variants in 21q11.2 (*ABCC13* pseudogene), 19p13.3 (*DAZAP1*), 1p36.33 (*CDK11B*, *CDK11A*) and 1p36.11 (*RCAN3*) [108]. Gialluisi et al. identified suggestive associations in 7q32.1 (*CCDC136*/*FLNC)* and 22q12.3 (*RBFOX2)* [109]. Others failed to find supportive evidence [103].

As mentioned earlier, in their latest report, Gialluisi et al. interrogated GWAS data from a very large sample of dyslexic cases and controls and apart from identifying *VEPH1* (3q25) as the top candidate gene, their analysis highlighted the association of dyslexia with ADHD, and an even stronger association with intelligence, bipolar disorder and schizophrenia [26], further supporting the notion of cross-disorder susceptibility between psychiatric and neurodevelopmental phenotypes. Of course, the hypothesis of a shared genetic background between dyslexia and ADHD, which occurs in approximately 25–40% of dyslexic individuals [169], has been a subject of extensive study. Comorbid cases exhibit more extensive and severe neuropsychological weakness and symptoms manifestation [170,171]. It was also shown that the heritability of reading disabilities was significantly higher in dyslexic individuals who also met criteria for ADHD [171]. Numerous recent studies support the SLD-ADHD common etiology hypothesis: Field et al. reported common loci implicated in both dyslexia and ADHD [67]. Mascheretti et al. found evidence for a *DCDC2* SNP (rs793862) via gene × gene interaction with *KIAA0319* with hyperactivity/impulsivity, a finding replicated in two independent samples [172], that was soon after also reported for the inattentive subphenotype [73].

Taking a step further, Verhoef et al. interrogated ADHD-related PRSs in relation to reading-related abilities in a large sample of children (~6000 individuals) from the UK Avon Longitudinal Study of Parents and Children (ALSPAC) in an effort to find evidence for shared genetic factors between ADHD and reading. Notably, polygenic ADHD risk was associated not only with reading but also with language-related abilities, further strengthening the hypothesis of shared genetic etiology between reading, language and ADHD [173]. In a GWAS study of ~2300 dyslexia cases and ~6300 controls, PRS analysis highlighted anew the correlation of ADHD with dyslexia and an even stronger association of dyslexia with two psychiatric disorders (schizophrenia and bipolar disorder) [26]. Price et al. performed a similar analysis starting from a GWAS on two children’s cohorts (~5250 individuals) aiming to explore the genetic architecture of reading; they used PRS from publicly available datasets on neurodevelopmental and psychiatric disorders and found a statistically significant association between ADHD and reading, as well as an overlap of 22 reading-associated genes previously implicated in ASD [157]. In fact, the relationship between dyslexia and ASD has not been extensively studied and data on the prevalence of ASD in cohorts ascertained for reading disabilities are most likely non-existent [174].

Despite preliminary evidence, however, it is too soon to say whether the observed shared genetic susceptibility between dyslexia and ADHD can be also reflected in brain’s disease-related anatomical structures and functional alterations. In two recent sMRI meta-analyses on grey matter differences in isolated ADHD versus dyslexia, no shared neural correlates were found [175,176]. On the other hand, when ADHD and dyslexia coexist, alterations (decreased cortical thickness) can be observed in brain regions relevant for both disorders, supporting the common etiology hypothesis; the same can be said for comorbid cases who exhibit reduced brain activity (during fMRI tasks) in regions associated with deficits in either isolated ADHD or dyslexia [176].

In Table 5 we provide the updated list of genes that have been, so far, implicated in different SLD domains, along with basic information on their biological role (Section 6). In parallel, we indicate which candidate SLD genes have shown association with other neurodevelopmental disorders, as curated in public databases (e.g., SFARI Gene database; [177]) and in literature.

## 5. Emerging Data from Neuroimaging Genetic Studies

Brain scans using modern technologies have provided ground-breaking insights into the workings of the human brain. Various MRI techniques have been most popularly used to visualize and explore: (a) structural abnormalities [e.g., cortical surface area (cSA) and cortical thickness; grey matter (GM) and white matter (WM) density and volumes] (sMRI), (b) alterations in structural connectivity between brain areas (DTI), and (c) functional abnormalities either in resting state or while performing (a) task(s) (reading-related, phonological, auditory, semantic, working-memory, visual-spatial, attentional, mixed) (fMRI).

Dyslexia has been associated with various anatomical and functional changes in the brain. In brief, total brain volume, GM and WM volume, total intracranial volume, cortical thickness and cSA, global and local brain asymmetries, level of gyrification, and to a lesser extent sulci configuration, have been under intensive research, not necessarily reaching an agreement regarding how these global brain measures are affected in dyslexia [15]. Regarding brain activity alterations, fMRI analyses show that cerebral hypoactivation seems to prevail over hyperactivity [37,178].

Interestingly, alterations seen in pre-reading children at risk for dyslexia are in agreement with results from children diagnosed with dyslexia [37]. This favors the idea that atypical brain development likely associated with dyslexia could be present within the first years of life and that dyslexia deficits may result from altered structural connectivity [179]. Moreover, faster WM development was observed in good versus poor readers from pre-reading to beginning-to-read and to fluent-reading stages, as well as a positive association between WM maturation and reading development [180]. Such data from neuroimaging studies in infants and pre-reading children, in concert with the high heritability estimates for reading abilities and disabilities, could suggest that dyslexia susceptibility genes may be involved in atypical neural migration and/or axonal growth during early (even in utero) brain development.

In the recently published, massive neuroimaging genetics meta-analysis study of the ENIGMA Consortium, it was shown that general cognitive function and educational attainment are the two cognitive traits that exhibit the most significant positive genetic correlation with cSA. According to the radial unit hypothesis, the expansion of cSA is driven by the proliferation of neural progenitor cells. Common variants explained 34% of the variation in total cSA; importantly, these variants have been associated with altered gene regulatory activity in neural progenitor cells during fetal development [181]. However, no GWAS and sMRI data from learning (dis)abilities and/or dyslexia studies were used in this meta-analysis, presumably because ENIGMA does not host an SLD working group.

Nevertheless, an extremely informative review on the neuroimaging genetics of dyslexia was published in 2017 by Mascheretti and co-workers; therein, the authors have done meticulous work to compile all available information from neuroimaging genetic association studies in established and candidate dyslexia genes, either in dyslexic cases or in the general population, covering studies published between 2010 and 2016 [37]. Thus, it is beyond the scope and the allocated space of the present article to review all dyslexia neuroimaging genetic studies anew. Instead, we have summarized findings published only in the last five years, with a focus on dyslexia and reading abilities (Table 4).

Among the most recent studies that led to the identification of novel dyslexia candidate genes, it is interesting to highlight that an intronic SNP located in *CEP63* was associated with WM volume in both right and left hemispheres of healthy individuals, as well as with reading comprehension scores [146]. The cluster of significant effect overlapped with a brain region previously found to be significant for SNPs within *DYX1C1* and *KIAA0319* [182]. Moreover, the right temporoparietal region associated with rs1064395 in *NCAN* and also overlapped with a region previously associated with the dyslexia susceptibility genes *KIAA0319*, *DYX1C1* and *MRPL19*, as well as *CEP63* [69,183]. The 15q11.2(BP1-BP2) deletion CNV, previously associated with a larger corpus callosum [43], was also associated with a smaller left fusiform gyrus as well as with altered activation; decreased activation was also observed for the left angular gyri, regions shown to associate with language and arithmetic tasks (Table 4) [44].

Pinel and Dehaene used fMRI to investigate heritability for brain activation while participants performed mental calculations. Posterior superior parietal lobules (SPL), right intraparietal sulcus (IPS), a left superior frontal region and left inferior parietal cortex (IPC) were under genetic influence [184]. Regarding dyscalculia, it was shown that dyscalculic children have decreased GM and WM volumes in the frontoparietal network, which might be associated with impaired arithmetic processing skills, whereas the WM volume decrease in parahippocampal areas may have an influence on fact retrieval and spatial memory processing [185,186]. Brain activation patterns of children with dyslexia, dyscalculia and comorbid dyslexia/dyscalculia were highly similar in how they deviated from neural activation patterns in control children when performing arithmetic tasks while undergoing fMRI [187]. Bulthe et al. recently revealed a significant deficit in number representations in temporal, parietal and frontal regions and a hyper-connectivity in visual brain regions in adults with dyscalculia [188].

Despite the progress in unravelling the polygenic nature of SLD, even with the latest molecular genomics approaches, combined with unprecedented technological advances in neuroimaging, we still lack a comprehensive and united understanding of SLD, whereas the field of neuroimaging genetics is in its infancy. One proposal to utilize neuroimaging genetics to identify biological causes of dyslexia would be to perform MRI imaging before the onset of reading acquisition, ideally in populations enriched with children at-risk of dyslexia (due to family history or parents or siblings with dyslexia). Given that the individual’s genetic makeup does not change in lifetime, a longitudinal design that would allow neuroimaging follow-up of these at-risk children until they reach reading (dis)abilities could be ideal in determining both the predictive role of brain scanning and the causal role of genetics. We reproduce this idea by Ramus et al. and expand it by adding genetics into the picture, yet we cannot but emphasize all the increased demands and challenges such a study design would impose [15]. However, it is of equally crucial importance to more deeply comprehend the neurobiology underlying these complex phenotypes and how established and emerging genes, and their variation, determine and affect neuronal development, respectively; we briefly touch on this subject in the following section.

**Table 4:** Recent (2015-presently) neuroimaging genetic studies reporting associations between genes and genomic loci associated with reading and mathematical (dis)abilities. The list is ordered based on evidence of association for genomic loci previously associated with SLD (that is, from replicated associations to newer evidence).

Phenotype (Trait/Subphenotype)	Gene (Associated Variant)	Association Outcome	Neuroimaging Technique	Reference (Population)
Dyslexia (poor reading comprehension)	*DCDC2* *READ1* element (RU2Short allele)	Higher R hemisphere connectivity: Stronger functional connectivity between R insula/IFG and R SMG	fMRI (resting state)	[189] (Hispanic- and African-Americans)
Dyslexia	*KIAA0319* (rs6935076)	Positive correlation between the number of minor alleles and the degree of neural variability in primary auditory cortex (cases and controls)	MEG	[190] (US population)
Typically developing children without mathematical training	*ROBO1* (9 SNPs)	GM pattern of the R parietal cortex (IPS and SPL)	sMRI	[191] (German population)
Dyslexia	*NRSN1* (3 SNPs) *FOXP2* *(6 SNPs)* *CNTNAP2* *(7 SNPs)* *CMIP* *(6 SNPs)*	*NRSN1*: GM volume in R dorsal parieto-occipital cortex, L lateral occipital cortex, L temporo-occipital fusiform cortex (visual word form area)/WM volume in L post-central cortex *FOXP2*: GM volume in L medial superior frontal gyrus *CNTNAP2:* WM volume in L cerebral and cerebellar peduncles *CMIP:* WM volume in R + L portions of cerebellum	sMRI	[192] (German population)
Dyslexia + Dyscalculia	15q11.2(BP1-BP2) (deletion CNV)	Smaller L fusiform gyrus (less GM) and less WM in R cerebellum, R paracentral lobule and L STL Decreased L fusiform and L angular gyri activation	sMRI fMRI	[44] (Icelandic population)
Reading comprehension scores	*CEP63* (rs7619451)	Increased WM volume in R + L hemisphere (temporoparietal region) of healthy individuals	sMRI	[146] (Swedish population)
Typically developing individuals	*NCAN* (rs1064395)	Increased WM volume in R + L temporoparietal and L inferior frontal brain regions (young adults) Increased GM volume in R + L cingulate, R superior frontal and R inferior parietal regions (infants)	sMRI	[69] (Finnish and Swedish population)
Typically developing individuals (reading ability) Brain activity (in 6 ROIs)—Typically developing children (phonological skills, reading competence)	*BDNF* (rs6265 or p.V66M)	Greater activation in reading- related regions (fusiform gyrus, L IFG, L STG) and greater activation in the hippocampus Increased brain activity in ROI 2 (bilateral hippocampus/parahippocampal gyrus/fusiform gyrus/cerebellum) and ROI 3 (L middle frontal gyrus/IFG/thalamus)	fMRI fMRI	[99] (US population—86.4% of Caucasian origin) [100] (US population—86.2% of Caucasian origin, or which 86.2% overlap with samples from [99])
Typically developing children and young adults (RAN)	rs1555839 (30kb upstream of *RNLS)*	Decreased cortical volume in the R IPL	sMRI	[110]

CNV: copy number variant, R: right, L: left, WM: white matter, GM: grey matter, fMRI: functional MRI, sMRI: structural MRI, STL: superior temporal lobe, SPL: superior parietal lobe; IPL: inferior parietal lobe, IPS: intraparietal sulcus, IFG: inferior frontal gyrus, MFG: middle frontal gyrus, STG: superior temporal gyrus, SMG: supramarginal gyri, *READ1*: regulatory element associated with dyslexia 1, ROI: region of interest, RAN: rapid automatized naming, MEG: magnetoencephalography.

## 6. A Glimpse on the Biological Background of SLD

The polygenic nature of SLD points to the existence of multiple causal pathways, much like most other neurodevelopmental disorders, where each variant contributes by a small effect to the total phenotypic variation. As observed via electrophysiological and neuroimaging studies in infants and pre-reading children, brain alterations predate reading ability or reading impairment, supporting the hypothesis that variants functioning in dyslexia susceptibility genes lead to atypical neural migration and/or axonal growth during early, most likely in utero, brain development [193,194].

**Figure 2:**
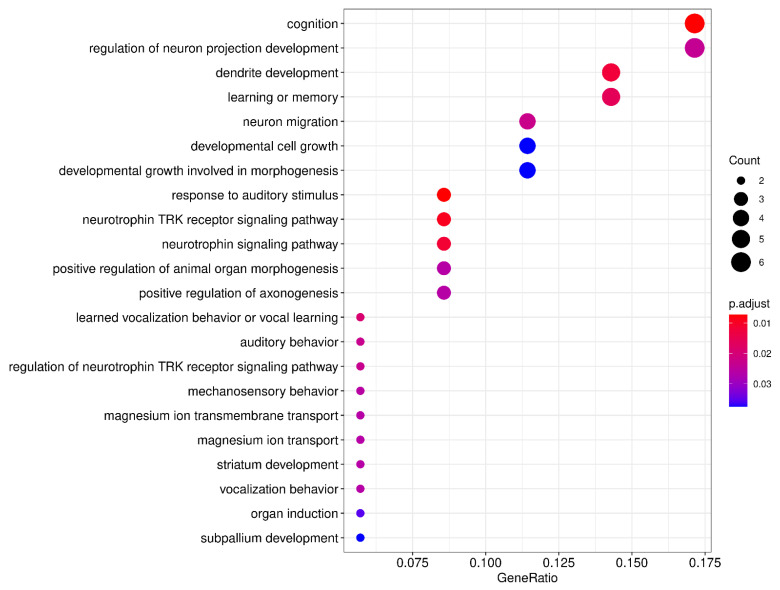
Top gene ontology (GO) terms (Biological Processes; *y*-axis) significantly enriched in the Figure 1. The figure was generated in R using *ClusterProfiler*. Count: Number of genes per GO term (gene-set), GeneRatio: Number of genes per GO term (gene-set) to the total number of queried genes (*n* = 36), p.adjust: Adjusted *p*-value using the Benjamini–Hochberg correction for multiple comparisons (*p* < 0.05). Data accompanying this figure are available in the Appendix A (Table A1).

However, the underlying neurodevelopmental causes of dyslexia are not fully understood. Original post-mortem neuroanatomical studies on dyslexia cases, conducted almost 35 years ago, were later followed by neuroimaging studies in humans and functional (knock-down and knock-out) animal studies. These studies lend support to the hypothesis that neuronal migration disturbances during development lead to misplacement of neurons, likely resulting in changes in white and grey matter [35,195]. The pathways that have emerged by now are relevant to neuronal migration and positioning, axon guidance regulating brain connectivity, dendritic growth, synaptic plasticity/transmission, cell adhesion, and sex hormone biology (Table 5) [36]. *ROBO1*, *KIAA0319*, *DCDC2*, *DYX1C1* gene products are mostly implicated in neurite outgrowth, neural connectivity, migration and development (Figure 2).

Although prior evidence from functional studies lend support to the idea that abnormal neuronal migration constitutes the neurobiological basis of dyslexia, which largely explains why this has been the most often cited hypothesis, in their recent review Guidi et al. advocate otherwise. The authors critically evaluated the hypothesis of neuronal migration and concluded that the evidence from histopathological and imaging studies in humans and functional studies in animal models is not robust enough to support it. The readers are encouraged to consult Table 1 from Guidi et al. for a thorough review on functional studies on key dyslexia genes conducted in several animal species and cell lines; therein, the authors have compiled data from reports in favor of the neuronal migration hypothesis as well as from studies refuting it [196].

Original studies also failed to find associations supporting the neuronal migration, axon guidance or steroid hormone-related pathways [75,104,109,128]. Thus, it emerges that although researchers have been keen to place many of the dyslexia candidate genes in a theoretical molecular/cellular model network involved in neuronal migration and neurite outgrowth, it seems unlikely that there is just a single explanatory model that connects all dyslexia-associated proteins on the molecular level. Rather, several etiological cascades contributing to dyslexia are likely to exist [35].

In fact, several reports have demonstrated that many dyslexia candidate genes, such as *DYX1C1* and *DCDC2*, have a reported structural or functional role in cilia [147,149,197]. Loss-of-function mutations in *DYX1C1* and *DCDC2* have been found in patients with ciliopathies: *DYX1C1* in cases of primary ciliary dyskinesia, with ciliary defects also confirmed in mouse and zebrafish models [148], and *DCDC2* in patients with nephronophthisis-related ciliopathy, inherited deafness and neonatal sclerosing cholangitis [150,151,152,153]. Conversely, we are unaware whether patients with such ciliopathies, caused by *DYX1C1* and *DCDC2* mutations, show symptoms of SLD or other cognitive impairments.

Other dyslexia candidate genes, such as *PCNT*, *CEP63* and *TUBGCP5*, are involved in centrosome and basal body biology (Table 5) [65,146,198]. *TUBGCP5*, PCNT and CEP63 are three of many centrosomal proteins involved in microtubule organization and even though they are ubiquitously expressed, brain-specific isoforms may be affected by rare variants. Centrosomal proteins are important in proper cell cycle progression; PCNT and CEP63 deficiencies were separately shown to cause microcephalic primordial dwarfism in humans [199,200], and a Seckel syndrome-like phenotype in mice, characterized by mitotic errors leading to p53-dependent neuronal progenitor cell death [201]. Bieder et al. used human iPSCs to derive a neuroepithelial stem cell line and showed that genes related to cilia were significantly enriched among genes upregulated during neuronal differentiation; importantly, a significant number of dyslexia-associated genes were detected by RNA-sequencing, of which seven, including *DYX1C1*, were upregulated, adding further support to the hypothesis of cilia dysregulation [202].

Left–right brain asymmetry defects have been proposed as an anatomical basis to neurodevelopmental disorders, such as ASD and dyslexia, possibly mediated by ciliary dysfunction [155]. Although the aforementioned proteins are associated structurally or functionally with primary cilia, microtubules and centrosomes, it remains unclear by which molecular mechanisms aberrations in their expression can lead to cognitive impairment. For an extensive presentation on the role of genes associated with cilia homeostasis/function and neurodevelopment/brain development, the readers are referred to excellent past reviews on the subject [36,155,203].

A gene’s expression or protein function is subject to genetic variation, and current methodologies allow us to observe this level of complexity with unprecedented detail by using genome-wide approaches. Still, genes do not act alone; they form pathways that interwind, creating higher-order networks and determining biological processes that are difficult to disentangle, especially in the case of complex traits lacking clear-cut diagnostic definitions, like dyslexia. Looking expectantly into the future, the ultimate goal for unravelling the biological mechanisms that contribute to and/or define SLD is the presymptomatic identification and development of age-adjusted precision intervention strategies, tailored to each individual’s language, educational demands and other social and psychological factors [110].

**Table 5:** Expression status in brain, cellular localization, and biological role of established and suspected genes associated with SLD susceptibility; the list is sorted by chromosome.

Chromosomal Locus ^1^	Gene ^2^	Gene Name	SLD Domain	Association with Other Neurodevelopmental Disorder(s) ^3^	Brain Expression Status ^4^	Subcellular Localization ^4^	Biological Role ^4^ (Protein Function, Biological Process)	Reference ^5^
1p34.3	*KIAA0319L*	KIAA0319 like	Dyslexia		Yes (Low specificity)	Nucleoli, plasma membrane, Golgi apparatus	Axon guidance—interaction with RTN4R	
2p12	*MRPL19*	Mitochondrial ribosomal protein L19	Dyslexia		Yes (Low specificity)	Mitochondrion	Ribosome biogenesis (39S subunit), rRNA processing Mitochondrial protein synthesis	[53]
2p12	*GCFC2* (*C2orf3*)	GC-rich sequence DNA-binding factor 2	Dyslexia		Yes (Low specificity)	Nucleoplasm, nucleolus	Pre-mRNA splicing, intron turnover and RNA processing	[205]
3p12.3	*ROBO1*	Roundabout guidance receptor 1	Dyslexia + Mathematical abilities	ASD	Yes (Low specificity)	Plasma membrane	Axon guidance receptor regulating connections between brain hemispheres Neuronal axon guidance receptor for SLIT1 and SLIT2 (rat, Drosophila)	[206]
3q22.2	*CEP63*	Centrosomal protein 63	Dyslexia		Yes (Low specificity)	Centrosomal	Cilium structure and function – centrosome duplication and cell cycle progression	
3q25.31-q25.32	*VEPH1*	Ventricular zone expressed PH domain containing 1	Dyslexia		Low	Nucleoplasm, nucleoli, cytosol	Interacts with TGF-β receptor type-1 (TGFBR1) and inhibits dissociation of activated SMAD2 from TGFBR1, impeding its nuclear accumulation and resulting in impaired TGF-β signaling. May also affect FOXO, Hippo and Wnt signaling	
4q28.1	*SPRY1*	Sprouty RTK signaling antagonist 1	Dyslexia		Yes (Low specificity)	Nucleoplasm, Golgi apparatus, cytosol, plasma membrane	Negative feedback regulators of growth factors signaling - inhibits the RTK-Ras-MAPK pathway (mouse)	[207,208]
5q31.3	*PCDHG*	Protocadherin gamma (gene cluster)	Dyslexia		Yes (Enhanced)	Plasma membrane	Neuronal cell adhesion – formation and maintenance of neural circuits	
5q31.2	*SPOCK1*	SPARC (osteonectin), cwcv and kazal like domains proteoglycan 1	Dyscalculia		Yes (Enhanced)	Extracellular (secreted to blood)	Mouse neurogenesis and post-injury axonal growth – Mouse embryonic development	
6p22.3	*NRSN1* *(VMP)*	Neurensin 1	Dyslexia		Yes (Enriched)	Plasma membrane, cytosol	Neural organelle transport, transduction of nerve signals, nerve growth. May play a role in neurite extension	
6p22.3	*DCDC2*	Doublecortin domain containing 2	Dyslexia + Mathematical abilities + Dysgraphia	ADHD, SLI	Yes (Low specificity)	Microtubules, mitotic spindle, centriolar satellite, cytosol	Embryonic neuronal migration (rat) Ciliary functions - Length and signaling of primary cilia in neurons (rat, C. elegans) Glutamatergic synaptic transmission (mouse)	[82,147,209,210]
6p22.3	*KIAA0319*	KIAA0319	Dyslexia	ADHD, SLI	Yes (Enriched)	Extracellular (secreted)	Embryonic neuronal migration Growth and differentiation of dendrites (rat) Inhibition of axon growth	[211,212,213]
7q31.1	*FOXP2*	Forkhead box P2		SLI, ASD, ADHD	Low in adult brain	Nucleoplasm	Transcriptional repressor - May also play a role in developing neural, gastrointestinal and cardiovascular tissues. Can act with CTBP1 to synergistically repress transcription. Plays a role in synapse formation by regulating SRPX2 levels. Involved in neural mechanisms mediating the development of speech and language.	
7q35	*CNTNAP2*	Contactin associated protein 2	Dyslexia	SLI, TS, ASD, ID, CD	Yes (Enhanced)	Plasma membrane	Cell adhesion (neurexin) participating in the organization of myelinated axons - localization of K^+^ channels within differentiating axons (rat) – axon potential propagation	[214,215]
7q32.1	*CCDC136*	Coiled-coil domain containing 136	Dyslexia		Yes (Enriched)	Golgi apparatus, plasma membrane	Acrosome formation in spermatogenesis and in fertilization (rat). Insufficient data about biological role in the CNS.	
7q32.1	*FLNC*	Filamin C	Dyslexia	Association trend for ADHD	Yes (Low specificity)	Plasma membrane, cytosol	Large actin-cross-linking protein (mouse). Insufficient data about biological role in the CNS	[109]
11p14.1	*BDNF*	Brain derived neurotrophic factor	Dyslexia		Yes (Enhanced)	Nuclear speckles, mitochondria, extracellular (secreted)	Activates signaling cascades downstream of NTRK2. Survival and differentiation of neuronal populations of CNS. (mouse-rat)	
12p13.1	*GRIN2B*	Glutamate ionotropic receptor NMDA type subunit 2B	Dyslexia	ASD	Yes (Enriched)	Plasma membrane, endosome, lysosome, cytoskeleton	Component of NMDA receptor (excitatory synaptic transmission) Neuronal pattern formation, channel function, formation of dendritic spines in hippocampal pyramidal cells	
12q13.12	*TEX49* *(LINC00935)*	Testis expressed 49	Word reading		Not detected	Intracellular	-	[157]
	*CCNT1*	Cyclin T1			Yes (Low specificity)	Nucleoplasm	Regulatory subunit of the cyclin-dependent kinase pair (CDK9/cyclin-T1) complex	
15q11.2	*TUBGCP5*	Tubulin gamma complex associated protein 5	Dyslexia + Dyscalculia	ASD, ID	Yes (Low specificity)	Centrosome, cytoplasm	Microtubule nucleation at the centrosome	
	*NIPA1*	NIPA magnesium transporter 1		ID	Yes (Enhanced)	Early endosome, plasma membrane	Mg^2+^ transporter (mouse, Xenopus)	
	*NIPA2*	NIPA magnesium transporter 2		ID	Yes (Low specificity)	Early endosome, Golgi apparatus, plasma membrane	Selective Mg^2+^ transporter (mouse, Xenopus)	
	*CYFIP1*	Cytoplasmic FMR1 interacting protein 1		ID	Yes (Low specificity)	Cytoplasm, perinuclear region	Actin-binding. Axon outgrowth. Formation of membrane ruffles and lamellipodia. (rat) Binds to the mRNA cap - translational repression activity of FMR1 in brain (mouse)	[141,216,217]
15q21.3	*DNAAF4* (*DYX1C1*)	Dynein axonemal assembly factor 4	Dyslexia + Mathematical abilities + Dysgraphia		Yes (Low specificity)	Plasma membrane, cytosol and nucleus	Embryonic neuronal migration (rat). Cilia structure and motility (mouse, zebrafish, human) Estrogen receptors regulation (rat)	[149,218,219]
15q21.2	*CYP19A1*	Cytochrome P450 family 19 subfamily A member 1	Dyslexia		Yes (Low specificity)	Endoplasmic reticulum membrane, mitochondria	A cytochrome P450 monooxygenase implicated in steroid hormone metabolism (sexual brain differentiation, synaptic plasticity, dendritic and axonal growth)	[66]
16q23.2-q23.3	*CMIP*	c-Maf inducing protein	Dyslexia	DD, ASD, ADHD, SLI	Yes (Low specificity)	Nucleoplasm, cytosol	T-cell signaling pathway	
17q21.31	*NSF*	N-ethylmaleimide sensitive factor, vesicle fusing ATPase	Dyslexia		Yes (Enhanced)	Golgi apparatus, cytosol	Hydrolase (substrates: ATP and H_2_O) Vesicle-mediated transport	
18p11.21	*MC5R*	Melanocortin 5 receptor	Dyslexia		No (human brain) Yes (mouse & pig)	Plasma membrane	G-protein coupled receptor for MSH and ACTH - possible mediator of the immunomodulation properties of melanocortins	
18q21.1	*DYM*	Dymeclin			Yes (Low specificity)	Golgi apparatus, cytoplasm, plasma membrane	Organization of Golgi apparatus Bone development	
18q21.31	*NEDD4L*	NEDD4 like E3 ubiquitin protein ligase			Yes (Low specificity)	Golgi apparatus, endosome, cytoplasm	Accepts ubiquitin (Ub) from an E2 Ub-conjugating enzyme and transfers Ub to targeted substrates. Inhibits TGF-β signalling. Ubiquitination and internalization of plasma membrane channels. Ubiquitination and degradation of SGK1 and TNK2. Ubiquitination of BRAT1. Dendrite formation by melanocytes. Regulator of TOR signalling. Ubiquitinates and regulates NTRK1protein levels.	
19p13.11	*NCAN*	Neurocan	Dyslexia		Yes (Enriched)	Extracellular (secreted in brain)	A chondroitin sulfate proteoglycan that binds to neuronal cell adhesion molecules and inhibits neuronal adhesion and neurite growth (chicken, rat)	[220,221]
21q22.3	*PCNT*	Pericentrin	Dyslexia	-	Yes (Low specificity)	Centrosome	Interacts with proteins involved in cilia assembly Component of filamentous matrix of the centrosome - microtubule network formation (nucleation) via anchoring γ-tubulin to centrosome Preventing premature centrosome splitting - inhibiting NEK2 kinase activity Interneuron migration	[222,223]
21q22.3	*DIP2A*	Disco interacting protein 2 homolog A	Dyslexia	ASD	Yes (Low specificity)	Plasma membrane, nucleoplasm, mitochondrion	Acetylation of CTTN - ensuring correct dendritic spine morphology and synaptic transmission (mouse)	
21q22.3	*S100B*	S100 calcium binding protein B	Dyslexia		Yes (Enriched)	Nucleoplasm, cytosol, extracellular region	Neurite extension and axonal proliferation (mouse) Binds calcium and zinc - modulates protein phosphatase 5 function	[224,225]
21q22.3	*PRMT2*	Protein arginine methyltransferase 2	Dyslexia		Yes (Low specificity)	Nucleoplasm, cytosol	Arginine methyltransferase Inhibits NF-kappa-B transcription (mouse). Coactivator for androgen and estrogen receptors	
22q12.1	*MYO18B*	Myosin XVIIIB	Mathematical abilities		Not detected	Νucleoplasm & centrosome (muscle cells and cardiomyocytes)	May regulate muscle-specific genes (nucleus) and may influence intracellular trafficking (cytoplasm)	
22q12.3	*RBFOX2*	RNA binding fox-1 homolog 2	Reading and language abilities		Yes (Low specificity)	Nucleoplasm, cytosol	Regulator of alternative splicing in neurons – Cerebellar development and physiology (mouse)	[109,226]
Xq21.31	*PCDH11X*	Protocadherin 11 X-linked	Dyslexia	ASD	Yes (Enriched)	Plasma membrane	Potential calcium-dependent cell-adhesion protein (mouse)	

ASD: Autism spectrum Disorder, ADHD: Attention Deficit/Hyperactivity Disorder, DD: Developmental Delay, ID: Intellectual Disability, TS: Tourette Syndrome, SLI: Specific Language Impairment, CD: Conduct Disorder. ^1^ Chromosomal loci are presented according to human genome assembly GRCh38.p13, obtained through Ensembl - Release 103 (February 2021). ^2^ Older gene nomenclature or synonyms are presented in parentheses. ^3^ Information on association with other neurodevelopmental disorders was obtained from SFARI Gene Database for ASD [177] taking into consideration all scoring levels (from high confidence to suggestive evidence) and ADHDgene database [227] which was last updated in February 2014. ^4^ Information for brain expression status, subcellular localization and biological role retrieved from ‘The Human Protein Atlas’ [228], and UniProt [229]. For annotation please refer to ‘The Human Protein Atlas’. ^5^ Reference provided in addition to information retrieved from ‘The Human Protein Atlas’.

## 7. Future Research Directions and Open Questions

It is of interest that increased frequency of sex chromosome aneuploidies (SCA) was reported among SLI and SLD individuals, albeit not statistically significant for the SLD group compared to the general population [230]. Individuals with SLI and SLD do not routinely undergo cytogenetic analysis, so their karyotype remains unknown. On the other hand, it is well-established that individuals with SCAs often show cognitive impairments, including speech and language, learning and mathematical disabilities [231,232]. At this point, it remains unclear whether the underlying biological defect for learning impairment in SCA cases is the deviation from X or Y chromosome gene(s) dosage alone, the co-inheritance of additional structural variations, such as CNVs [233], specific changes in brain anatomy affecting cognition [234], or a combination of those. Overall, these data highlight the importance of combinatorial evaluation of such neurodevelopmental phenotypes that can benefit from early detection and appropriate management, especially considering the large proportion of cases with SCAs that remain undiagnosed [235].

Another open question is why SLD seems to be more prevalent in males than in females worldwide [236,237,238,239,240], as it is also observed for other neurodevelopmental disorders [241,242,243]. SLD sex ratios range from about 1.5–3.3:1 in epidemiological samples to 3:1 to 5:1 in referred samples [239]. If this universal sex bias cannot be attributed to factors such as ascertainment bias, definitional or measurement variation, severity of disability, language transparency and alphabet, educational practices or unequal opportunities, race, or socioeconomic status [1,9,244], then what is the remaining underlying causal factor? Arnett et al. suggested that it could be partially explained by cognitive correlates emerging prior to schooling, such as reading ability (slower processing speed in males), which could serve as a proxy for the sex difference in brain development [239]. From the biological perspective, however, convincing genetic evidence to explain the sex bias observed in SLD is still lacking or is at least contradictory [15]. In one twin study, males had greater heritability estimates (*h*^2^) than females in word recognition deficit [245], whereas in another the sex-specific *h*^2^ estimates did not reach statistical significance [246]. In a Chinese cohort of dyslexic children and adolescents analyzed for *CNTNAP2*, two common variants were found to confer protection against dyslexia in females; one of these variants was marginally associated with the environmental factor of scheduled reading time in female homozygotes showing lower risk for dyslexia [247]. This type of associations will require extensive approaches on genome-wide level, before we begin to speculate which molecular mechanisms underlie sex-specific brain functions. According to the liability threshold model [248], females who meet a diagnostic threshold for ASD or ADHD are expected to carry a higher genetic burden than males and male relatives of females with ASD or ADHD are more likely to be also affected than relatives of affected males [241,249]. To date, although being the subject of great debate, it remains unclear whether hormonal, genetic, epigenetic, cognitive, neurological, anatomical or environmental factors or combinations of the above contribute to sex-biased susceptibility to any of the aforementioned disorders [250], including SLD.

## 8. Conclusions

In the quest for unraveling the genetic architecture of as complex a phenotype as SLD, various methodological approaches have been applied since the first dyslexia-associated genes were identified back in the 1990s (Table 1). In the time that lapsed since, classical linkage studies in unique, large pedigrees—segregating rare, private mutations—chromosomal aberrations, genetic associations, and lately large-scale high-throughput genome-wide genotyping and sequencing studies (Table 2 and Table 3) have continued to shape our understanding of this highly complex disorder. The list is continuously populated with novel gene associations whose protein products participate in a variety of biological processes (Table 5). Whether their relevance to SLD manifests via alterations in brain anatomy, connectivity and function (assessed via neuroimaging techniques—Table 4) or via perturbed cellular mechanisms (assessed via functional studies) raises the need for more research in order to reach confidence that these associations hold true. The nature of SLD, unique to our humankind and to properties of the human brain, renders the in vivo experimentation in other species suboptimal. With new technologies and analytical tools, including fourth-generation sequencing and neuroimaging, we will continue to search for the missing heritability with the ultimate hope that at least some genetic findings will translate into predictive and/or preventive measures. To do so, we will need to bridge the knowledge gaps between genomics, molecular pathways, cellular communication, neuronal circuits, neuroimaging data, with human cognition and brain function. This is a long but intriguing path to take for scientists approaching SLD from different scientific disciplines, yet ‘intriguing’ has always been the driving force.

## Data Availability

No new data were created or analyzed in this study. Data sharing is not applicable to this article.

## Appendix A

**Table A1:** Original data pertaining to Figure 2.

ID	Description	Gene Ratio	BgRatio	p Value	p.Adjust	q Value	Gene ID	Count
GO:0010996	response to auditory stimulus	3/36	26/18,670	1.66 × 10^−5^	0.0086304564034056	0.00703130190039533	*KIAA0319/FOXP2/CNTNAP2*	3
GO:0050890	cognition	6/36	296/18,670	1.97 × 10^−5^	0.0086304564034056	0.00703130190039533	*FOXP2/CNTNAP2/BDNF/GRIN2B/CYFIP1/S100B*	6
GO:0048011	neurotrophin TRK receptor signaling pathway	3/36	33/18,670	3.45 × 10^−5^	0.0100801981306244	0.00821241808767524	*SPRY1/BDNF/CYFIP1*	3
GO:0038179	neurotrophin signaling pathway	3/36	39/18,670	5.74 × 10^−5^	0.012563268955858	0.0102353957607206	*SPRY1/BDNF/CYFIP1*	3
GO:0016358	dendrite development	5/36	233/18,670	7.97 × 10^−5^	0.0139693179256901	0.0113809151088896	*DCDC2/KIAA0319/CYFIP1/NEDD4L/RBFOX2*	5
GO:0007611	learning or memory	5/36	256/18,670	0.000124138944672079	0.0181242859221235	0.0147660007873104	*FOXP2/CNTNAP2/BDNF/GRIN2B/S100B*	5
GO:0098598	learned vocalization behavior or vocal learning	2/36	10/18,670	0.000161100993018104	0.020160638554837	0.0164250335738759	*FOXP2/CNTNAP2*	2
GO:0001764	neuron migration	4/36	157/18,670	0.000229876442023158	0.0243726689138727	0.0198566084157723	*SPOCK1/DCDC2/KIAA0319/DNAAF4*	4
GO:0031223	auditory behavior	2/36	13/18,670	0.000278226814085305	0.0243726689138727	0.0198566084157723	*FOXP2/CNTNAP2*	2
GO:0051386	regulation of neurotrophin TRK receptor signaling pathway	2/36	13/18,670	0.000278226814085305	0.0243726689138727	0.0198566084157723	*SPRY1/CYFIP1*	2
GO:0010975	regulation of neuron projection development	6/36	499/18,670	0.000348456394019227	0.0272748656682451	0.0222210513375032	*ROBO1/SPOCK1/KIAA0319/BDNF/CYFIP1/NEDD4L*	6
GO:0007638	mechanosensory behavior	2/36	15/18,670	0.000373628296825275	0.0272748656682451	0.0222210513375032	*FOXP2/CNTNAP2*	2
GO:1903830	magnesium ion transmembrane transport	2/36	16/18,670	0.000426486056377473	0.0279522417733895	0.0227729150713267	*NIPA1/NIPA2*	2
GO:0015693	magnesium ion transport	2/36	17/18,670	0.000482764901757518	0.0279522417733895	0.0227729150713267	*NIPA1/NIPA2*	2
GO:0110110	positive regulation of animal organ morphogenesis	3/36	81/18,670	0.000506610816906104	0.0279522417733895	0.0227729150713267	*ROBO1/SPRY1/FOXP2*	3
GO:0021756	striatum development	2/36	18/18,670	0.000542452180533815	0.0279522417733895	0.0227729150713267	*FOXP2/CNTNAP2*	2
GO:0071625	vocalization behavior	2/36	18/18,670	0.000542452180533815	0.0279522417733895	0.0227729150713267	*FOXP2/CNTNAP2*	2
GO:0050772	positive regulation of axonogenesis	3/36	85/18,670	0.000583381347572954	0.0283912255818838	0.0231305586932435	*ROBO1/BDNF/CYFIP1*	3
GO:0001759	organ induction	2/36	22/18,670	0.000815033760170356	0.0375773459952227	0.030614564509446	*ROBO1/SPRY1*	2
GO:0021544	subpallium development	2/36	24/18,670	0.00097144865957663	0.0418284073790138	0.0340779382395714	*FOXP2/CNTNAP2*	2
GO:0048588	developmental cell growth	4/36	234/18,670	0.0010340175426981	0.0418284073790138	0.0340779382395714	*BDNF/CYFIP1/NEDD4L/PRMT2*	4
GO:0060560	developmental growth involved in morphogenesis	4/36	235/18,670	0.00105048511682455	0.0418284073790138	0.0340779382395714	*SPRY1/BDNF/CYFIP1/NEDD4L*	4
GO:0021987	cerebral cortex development	3/36	116/18,670	0.00143700209694573	0.0547310363880202	0.0445898133514512	*ROBO1/FOXP2/CNTNAP2*	3
